# The role of prescription drugs in female overactive bladder syndrome—A population‐wide cohort study

**DOI:** 10.1002/pds.4920

**Published:** 2019-12-05

**Authors:** Wolfgang Umek, Andreas Gleiss, Barbara Bodner‐Adler, Berthold Reichardt, Christoph Rinner, Georg Heinze

**Affiliations:** ^1^ Department of Obstetrics and Gynecology Medical University of Vienna Vienna Austria; ^2^ Section for Clinical Biometrics, Center for Medical Statistics, Informatics, and Intelligent Systems (CeMSIIS) Medical University of Vienna Vienna Austria; ^3^ Sickness Fund Burgenland Burgenlaendische Gebietskrankenkasse Eisenstadt Austria; ^4^ Karl Landsteiner Institute of Specialised Gynecology and Obstetrics Medical University of Vienna Vienna Austria

**Keywords:** drug interactions, drug utilization, overactive bladder, pharmacoepidemiology, polypharmacy

## Abstract

**Purpose:**

Overactive bladder (OAB) syndrome has severe effects on quality of life. Certain drugs are known risk factors for OAB but have not been investigated in a population‐wide cohort. The objective of this study was to investigate the role of prescription drugs in the etiology of the OAB.

**Methods:**

Retrospective cohort study using a population‐wide database of 4 185 098 OAB‐naïve women followed Strengthening the Reporting of Observational Studies in Epidemiology guidelines. We investigated the subscription use of anticholinergic medication and 188 chemical substances, which are suspected triggers for OAB (trigger medications [TMs]). We hypothesized a relationship between the prescription for one or more TM and the prescription for anticholinergic medication against OAB (marker medication [MM]).

**Results:**

The use of MM in Austria increased from 2009 to 2012 on average by 0.025 percentage points per year (95% confidence interval [CI]: 0.015‐0.036). In December 2012, 1 in 123 women filled a prescription for any MM, equaling an average utilization of 0.84%. The relative risk of filling a prescription for a MM 6 months after filling a prescription for a TM was 2.70 (95% CI: 2.64‐2.77). All investigated medication classes showed a higher risk for the prescription for MM. Medication from classes “genitourinary system and sex hormones” and “systemic anti‐infectives” caused the highest increase in risk (109% and 89%, respectively). Prescriptions for class “cardiovascular system” caused the lowest increase in the risk (15%).

**Conclusion:**

Certain prescription medications are a significant risk factor for the need to take anticholinergic medication as a consequence.

## 1 INTRODUCTION

**Key Points**

- Overactive bladder (OAB) syndrome is a form of lower urinary tract dysfunction with severe effects on quality of life, affecting 17% of the female population.
- The use of certain prescription drugs (trigger medication [TM]) ranks among the most important known risk factors for OAB.
- The use of prescribed medication against OAB syndrome (trospium chloride, oxybutynin, tolterodine, and solifenacin) increased in Austria from January 2009 to December 2012 on average by 0.025 percentage points per year (95% confidence interval [CI] 0.015‐0.036).
- The relative risk with respect to the need for prescription for medication against OAB syndrome was 2.70 (95% CI: 2.64‐2.77) half a year after the prescription for TM.
- Prescriptions for medication from class “genitourinary system and sex hormones” and “anti‐infectives for systemic use” cause the highest increase in risk for the prescription for medication against OAB (by 109% and 89%, respectively).

Overactive bladder (OAB) syndrome is a form of lower urinary tract dysfunction, characterized by urinary urgency, frequency, and nocturia, with or without urinary incontinence.^1^ Epidemiological data estimate the prevalence of the OAB at 17% in Austria,^2^ in line with the estimated prevalence of 13%‐16% of the female population in international studies.^3^ Quality of life is severely reduced in 66% of affected women as shown in a large epidemiologic study from Norway.^4^

The most important known risk factors for OAB are increasing age, obesity, presence of pelvic organ prolapse, parity, and the use of certain prescription drugs.^5^

For example, polyuria is a common side effect of loop diuretics, possibly leading to elevated urinary frequency and urgency.^6^ Nonsteroidal anti‐inflammatory drugs may lead to exacerbation of nocturia by redistributing body fluids.^7^ Acetylcholinesterase inhibitors have cholinergic activity, leading to OAB symptoms.^8^ Calcium channel blockers are associated with a malfunction of adequate relaxation and emptying of the bladder. The daily use of oral estrogens is considered to worsen urinary incontinence; however, its pathophysiology remains unknown.^9^ Antidepressants, antipsychotics, and benzodiazepines drugs, which impact on the central nervous system, have also been shown to trigger the development of OAB.^10^, ^11^ Their effects might result from relaxing the pelvic floor muscles, interference with afferent nerve pathways from the bladder, or from indirectly leading to an inability to toilet.

OAB and its impact on quality of life are probably more pronounced in the elderly, a population already impaired by other medical comorbidities and vulnerable to the side effects of medications.^12^ Previous studies, including those of our own research group for patient cohorts not restricted by age, have shown that patients are often overtreated with prescription drugs. Heinze et al reported a prevalence of 13%‐15% of double medication with antihypertensive, lipid‐lowering, or hypoglycemic drugs in a population‐wide study.^13^

The role of prescription drug utilization in the etiology of the OAB has not yet been investigated using a rigorous scientific approach applied to a population‐wide cohort. In order to reduce medication risks in OAB patients, a greater understanding of the prevalence and use of these prescription medications is necessary.

The objective of this study was firstly to describe the prevalence of filling a prescription for anticholinergic medication (marker medication [MM] for OAB over a 4‐year period), and secondly to investigate the role of specific prescription drugs in the etiology of the OAB by means of a population‐wide cohort study.

## 2 METHODS

### 2.1 Study design

We conducted a retrospective cohort study using a large population‐wide database. We followed the Strengthening the Reporting of Observational Studies in Epidemiology (STROBE) guidelines in analyzing and reporting our data (File S1).

### 2.2 Database

A large, comprehensive data set, provided by 13 Austrian social security institutions, covering over 9 million insured persons in Austria had already served for studying double medication.^13^ As social insurance is mandatory in Austria, this database holds population‐wide pseudonymized records of all drug prescriptions filled over several years. This database was used to evaluate a possible correlation of utilization of specific drug classes with the incidence of OAB syndrome.

### 2.3 Study population

The study population consisted of 4 185 098 insured and OAB‐naïve women without age limit in Austria who, during the time period of January 2009 to December 2012, had a contract with one of the nine provincial sickness funds or one of the four nationwide social security institutions for independent entrepreneurs, farmers, federal employees, and railway or mining employees.

### 2.4 Primary hypothesis

We hypothesized that in Austrian women of any age, there could be a relationship between the prescription for one or more trigger medications (TMs) and the prescription for MM for OAB syndrome.

A basic assumption of the study was that the prescription filling for any of the following four anticholinergic drugs indicated OAB syndrome in a woman: trospium chloride (Anatomical Therapeutic Chemical [ATC] code: G04BD09), oxybutynin (G04BD04), tolterodine (G04BD07), and solifenacin (G04BD08). Mirabegron (G04BD12) was not yet released in Austria during the study period, and no other drug to treat OAB was licensed. We defined the four aforementioned drugs as MM for the presence of OAB.

### 2.5 Data base

We devised a list of 188 chemical substances according to ATC Classification System level 5, in 13 anatomical main groups (ATC level 1), which are suspected to trigger the development or deterioration of OAB. ATC is a World Health Organization (WHO) controlled pharmacologic coding system, which lists all active ingredients of drugs according to the organ system which they act on.^14^ The inclusion of a chemical substance into the list was based on a review of the scientific literature^6^, ^7^, ^8^, ^9^, ^10^, ^11^, ^15^, ^16^, ^17^, ^18^, ^19^, ^20^, ^21^, ^22^, ^23^, ^24^, ^25^ and a cross‐check with Clinical Pharmacology, an online pharmacologic database.^26^ We defined these substances as “TM.” The substances that were tested for their role as TM are shown in their respective drug class according to ATC in Table 1.

**Table 1:** List of all substances, which are suspected to trigger OAB syndrome (TM), grouped according to ATC level 1

Substance class	ATC level 1	Substance (corresponding to ATC‐level 5)
Alimentary tract and metabolism	A	Amphotericin B, dexamethasone, metronidazole, metoclopramide
Blood and blood‐forming organs	B	Abciximab
Cardiovascular system	C	Carvedilol, dexamethasone, doxazosin, eprosartan, lidocaine, methyldopa, tetracaine
Dermatologicals	D	Antibiotics for dermatologic use, dexamethasone, lidocaine, lithium, methylprednisolone, metronidazole, tacrolimus, tetracaine
Genitourinary system and sex hormones	G	Amphotericin B, bromocriptine, chlormadinone, desogestrel, dienogest, drospirenone, estradiol, estriol, ethinylestradiol, etynodiol, gestodene, metronidazole, naproxen, levonorgestrel, lynestrenol, medroxyprogesterone, megestrol, nomegestrol, norelgestromin, norethisterone, norgestimate, norgestrel, quingestanol, sildenafil, testosterone
Systemic hormonal preparations, excluding sex hormones and insulins	H	Dexamethasone, methylprednisolone
Anti‐infectives for systemic use	J	Amphotericine B, cidofovir, itraconazole, meropenem, metronidazole, voriconazole
Antineoplastic and immunomodulation agents	L	Arsenic trioxide, belatacept, bexarotene, bortezomib, ciclosporin, cytarabine, ethinylestradiol, goserelin, ifosfamide, interferon beta‐1a, leuprorelin, mycophenolic acid, natalizumab, nilotinib, peginterferon beta‐1a, polystradiol phosphate, tacrolimus, temozolomide
Musculoskeletal system	M	Baclofen, botulinum toxin, dantrolene, esomeprazole, febuxostat, misoprostol, naproxen
Nervous system	N	Acamprosate, aminobutyric acid, aripiprazole, bromocriptine, bupivacaine, buprenorphine, citalopram, clomipramine, COMT inhibitor, decarboxylase inhibitor, desvenlafaxine, donepezil, entacapone, escitalopram, felbamate, fentanyl, fluoxetine, gabapentin, ginko folium, lidocaine, memantine, mepivacaine, lamotrigine, lanzapine, levobupivacaine, levodopa, lithium, mirtazapine, naratriptan, olanzapine, paliperidone, paroxetine, pramipexole, pregabalin, quetiapine, rasagiline, remifentanil, riluzole, risperidone, ropinirole, rufinamide, selegiline, sertraline, tetracaine, tiagabine, tolcapone, trazodonevalproic acid, venlafaxine, ziprasidone, zonisamide
Antiparasitic products, insecticides, and repellents	P	Metronidazole
Respiratory system	R	Cetirizine, desloratadine, dexamethasone, levocetrizine, lidocaine, loratadine
Sensory organs	S	Ciclosporin, dexamethasone, lidocaine, methylprednisolone, tetracaine, travoprost

Abbreviations: ATC, Anatomical Therapeutic Chemical Classification System; OAB, overactive bladder; TM, trigger medication.

Basic variables included year of birth, sex, area of residence (zip code), date of deregistration from social security institution (if applicable), and date of death (if applicable). We studied all women without any limitations of their age. We accessed all prescriptions filled by insured women and recorded: number of dispensed packages, package size (number of pills per package), strength (dose per pill), pharmacy article identifier of the dispensed drug, and copayment waiver status.

Using a separate database, provided by the Austrian Agency for Health and Food Safety, we linked pharmacy article identifiers to the WHO fifth‐level, seven‐digit ATC codes. This database also contained information about all hospitalizations of all insured women during the study period, described by admission date, length of stay, and discharge diagnoses according to the International Classification of Diseases (ICD) by the WHO.

All variables could be retrieved completely; no missing data occurred.

### 2.6 Data protection

Data were fully pseudonymized using irreversible algorithms. Data were saved on a secured, access‐restricted database server provided by the IT Department of the Medical University of Vienna. Only authorized project members (G.H., A.G., and C.R.) were granted access to the database.

Permission for the study was granted by the authorized representative body of all 13 involved social security institutions and the ethics committee of the Medical University of Vienna (EK 1471/2016). Patients or patients' representatives were not involved in the planning or analysis of this study.

### 2.7 Statistical analysis

For each month of the years 2009‐2012, a prevalence of OAB was estimated as the number of women who filled a prescription for MM in the respective month divided by the number of women who filled any prescription in this month (births and deaths in a month were counted as 1/2 in numerator and denominator). This calculation from incidence to prevalence is based on the assumption that, on average, the MM was taken for 1 month after filling the prescription. Prevalences are graphically presented by locally weighted scatterplot smoothing (LOESS) curves and statistically tested for nonzero slope using simple linear regression models.

For the investigation of a potential influence of certain drugs on OAB, we included all women who had filled any prescription in the years 2009‐2012 but had not filled a prescription for any MM for the use against OAB in 2011 and in the first half of 2012. We called them “OAB‐naïve.”

The distribution of age is presented as median, quartiles, and range and graphically displayed using histograms.

Poisson regression models are used to quantify the influence of filling at least one prescription for TMs in the first half of 2012 on filling at least one prescription for MM in the second half of 2012. This results in estimates of relative risks (RRs) which directly translate into cost multipliers. We called the first half of 2012 the “harvesting period” and the second half of 2012 the “target period.”

The following variables were used as adjustment variables: filling a prescription for non‐TM of the same ATC class as the investigated TM, age, indicator of hospitalization (ie, admission to hospital for at least 1 day), 43 indicators, each corresponding to the ICD code of one medical condition associated with OAB as obtained from discharge diagnoses (Table 2). A number of 15 922 women (0.38%) who died in the second half of 2012 were downweighted in the regression model according to their proportional time at risk. Crude RRs as well as adjusted RRs with 95% confidence intervals (CIs) are presented.

**Table 2:** ICD‐10 codes of medical conditions assumed to be associated with OAB syndrome obtained from discharge diagnoses in the study population of 4 185 098 women (number and percent refer to first half of 2012)

ICD‐10 code	Medical condition	Number	%
E10	Type 1 diabetes mellitus	2505	0.06
E11	Type 2 diabetes mellitus	20 977	0.50
E12	Malnutrition‐related diabetes mellitus	372	0.01
E13	Other specified diabetes mellitus	560	0.01
E14	Unspecified diabetes mellitus	4754	0.11
E66	Obesity	12 911	0.31
F00	Dementia in Alzheimer disease	1857	0.04
F01	Vascular dementia	2798	0.07
F02	Dementia in other diseases classified elsewhere	199	<0.01
F03	Unspecified dementia	5796	0.14
F05	Delirium, not induced by alcohol and other psychoactive substances	806	0.02
F06	Other mental disorders due to brain damage and dysfunction and to physical disease	652	0.02
F17	Mental and behavioral disorders due to use of tobacco	5243	0.13
F32	Depressive episode	14 705	0.35
F33	Recurrent depressive disorder	5095	0.12
F34	Persistent mood disorder	723	0.02
F41	Other anxiety disorders	3439	0.08
H81	Disorders of vestibular function	3030	0.07
H82	Vertiginous syndromes in diseases classified elsewhere	22	<0.01
I50	Heart failure	11 933	0.29
I09	Other rheumatic heart diseases	14	<0.01
I20	Angina pectoris	3227	0.08
I21	Acute myocardial infarction	3004	0.07
I24	Other acute ischemic heart diseases	427	0.01
I25	Chronic ischemic heart disease	19 888	0.48
M00	Pyogenic arthritis	199	<0.01
M01	Direct infections of joint	8	<0.01
M02	Reactive arthropathies	98	<0.01
M03	Postinfective and reactive arthropathies	1	<0.01
M07	Psoriatic and enteropathic arthropathies	38	<0.01
M09	Juvenile arthritis	3	<0.01
M10	Gout	350	0.01
M13	Other arthritis	879	0.02
M15	Polyarthrosis	1491	0.04
M17	Gonarthrosis	9480	0.23
M19	Other arthrosis	3739	0.09
M25	Other joint disorders, not elsewhere classified	2714	0.06
N39	Other disorders of urinary system (including bacteriuria, infection, incontinence, and so on)	15 544	0.37
O23	Infections of genitourinary tract in pregnancy	490	0.01
O24	Diabetes mellitus in pregnancy	1152	0.03
Q24	Other congenital malformations of heart	62	<0.01
R42	Dizziness and giddiness	4705	0.11

Abbreviations: ICD, International Classification of Diseases; OAB, overactive bladder.

The same way of analysis was repeated for women of at least 65 years of age, as the subgroup of elderly patients is discussed in the literature.

All computations were carried out using SAS software Version 9.4 (SAS Institute Inc., Cary, NC, 2012).

### 2.8 RESULTS

In 2011, the Government of Austria reported 4 296 293 women living in Austria.^27^ Our data set for this study comprised a total of 4 185 098 OAB‐naïve women, that is, women who had filled any prescription in the years 2009‐2012, but had not filled a prescription for a MM against OAB syndrome in 2011 and the first half of 2012. Of these 4 185 098 OAB‐naïve women, 27 689 (0.7%) filled a prescription for MM in the second half of 2012. These 27 689 women were first‐time users of MM or newly diagnosed with OAB, and 4 157 409 OAB‐naïve women (99.3%) did not fill a prescription for MM in the same time.

**Figure 1:**
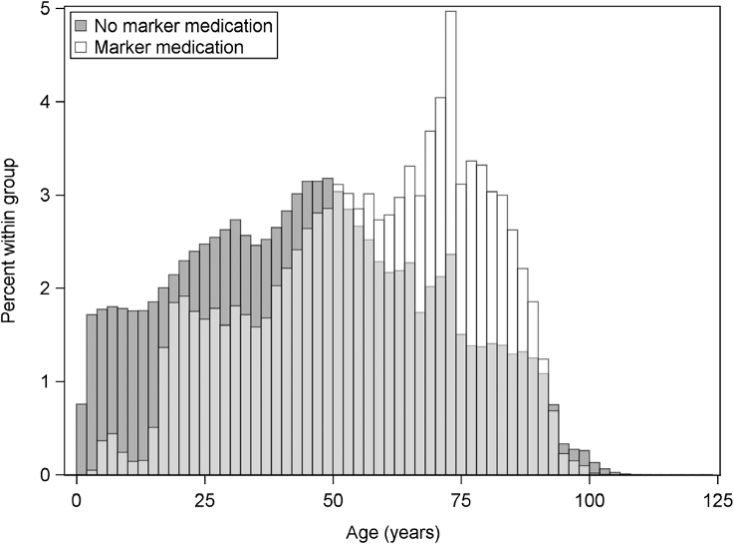
Distribution of age in women who filled prescriptions for MMs against OAB syndrome (white columns) compared to women who did not fill prescriptions for MMs (dark‐gray columns); overlap of groups (light‐gray columns). MM, marker medication; OAB, overactive bladder

Women who filled prescriptions for MM were older than women who did not fill prescriptions for MM; median age: 59 years (quartiles: 42‐73, range: 2‐103) compared to median age: 44 years (quartiles: 26‐63, range: 1‐124), see Figure 1; 429 751 women (10.27% of the study population) were hospitalized in the first half of 2012. The number and proportion of women with medical conditions associated with OAB are summarized in Table 2.

**Figure 2:**
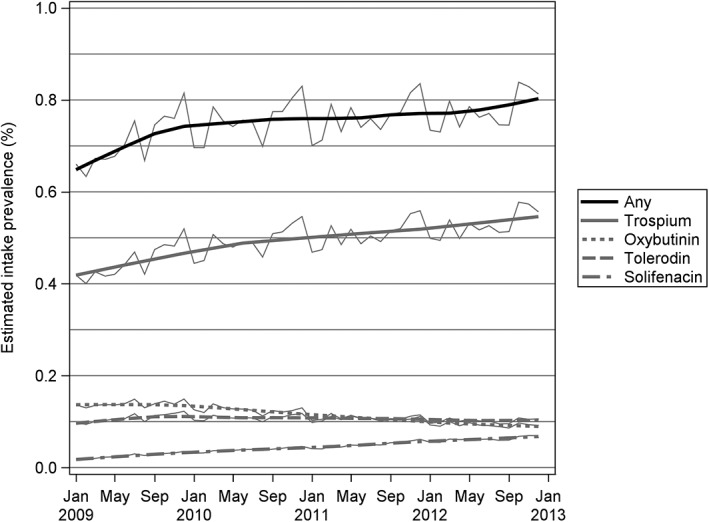
Temporal change of prescriptions for anticholinergic medication against OAB syndrome in Austrian women. Thin lines: observed values; thick lines: LOESS smoothed. LOESS, locally weighted scatterplot smoothing; OAB, overactive bladder

The filling of a prescription for any MM increased from January 2009 to December 2012 on average by 0.025 percentage points per year (95% CI = 0.015‐0.036; see Figure 2); 27 377 of 4 145 058 or 1 in 151 women filled any prescription for MM in January 2009, equaling an average utilization of 0.66%. In December 2012, 33 499 of 4 120 263 or 1 in 123 women filled a prescription for any MM, equaling an average utilization of 0.84%.

In particular, the filling of prescriptions for trospium increased from 17 364 of 4 145 058 (average utilization 0.42%) to 22 932 of 4 120 263 (average utilization 0.56%), the filling of prescriptions for solifenacin increased from 721 of 4 145 058 (average utilization 0.02%) in January 2009 to 2858 of 4 120 263 in December 2012 (average utilization 0.06%). The filling of prescriptions for tolterodine remained approximately constant during the observation period. The filling of prescriptions for oxybutynin decreased from 0.14% in January 2009 to 0.11% in December 2012. The temporal change of filling of prescriptions for MMs trospium, solifenacin, tolterodine, and oxybutynin is shown in Figure 2.

Of 901 924 women filling prescriptions for “TM,” that is, medication suspected to cause OAB syndrome during the first half of 2012, 1.31% had filled a prescription for any MM in the second half of 2012. Of women without TM, only 0.48% had filled a prescription for MM. The resulting RR was 2.70 (95% CI = 2.64‐2.77). The RRs and associated 95% CIs with respect to filling a prescription for a drug against OAB (MM) depending on the filling of a prescription for a TM by ATC classes of TM are shown in Table 3.

**Table 3:** RR with respect to the need of medication against OAB syndrome for taking versus not taking any medication from the indicated class (ATC level 1)

Medication class ATC level 1		Women without TM^a^		Women with TM^a^			95% CI crude RR			95% CI adjusted RR	
		Number	% with MM^b^	Number	% with MM^b^	RR crude	Lower limit	Upper limit	RR adjusted	Lower limit	Upper limit
A	Alimentary tract	4 148 711	0.65	36 387	1.91	2.93	2.72	3.16	1.40	1.29	1.51
C	Cardiovascularsystem	4 019 236	0.62	165 862	1.58	2.53	2.44	2.64	1.15	1.10	1.20
D	Dermatologicals	41 21 445	0.65	63 653	1.09	1.67	1.55	1.80	1.43	1.33	1.54
G	Genitourinary	4 087 060	0.63	98 039	1.88	2.97	2.83	3.11	2.09	1.99	2.19
H	Hormones	4 158 666	0.66	26 432	1.44	2.20	1.99	2.43	1.46	1.32	1.61
J	Anti‐infectives	4 170 304	0.66	14 794	1.36	2.06	1.80	2.37	1.97	1.71	2.26
L	Antineoplastics	4 176 834	0.66	8264	1.28	1.94	1.61	2.35	1.52	1.26	1.85
M	Musculoskeletal	4 095 534	0.65	89 564	1.37	2.11	2.00	2.24	1.60	1.51	1.69
N	Nervous system	3 727 019	0.56	458 079	1.53	2.75	2.68	2.82	1.61	1.56	1.66
P	Antiparasitic	4 163 505	0.66	21 593	1.29	1.96	1.75	2.21	1.89	1.68	2.12
R	Respiratory system	3 992 177	0.64	192 921	1.02	1.59	1.51	1.66	1.42	1.35	1.49
S	Sensory organs	4 180 249	0.66	4849	1.48	2.25	1.79	2.83	1.10	0.87	1.39

*Note*: Shown are crude and adjusted RRs including 95% CI, see Section 2.7 for a list of adjustment variables.

Abbreviations: CI, confidence interval; MM, marker medication; OAB, overactive bladder; RRs, relative risks; TM, trigger medication.

a Within first half of 2012.

b Within second half of 2012. MM, that is, anticholinergic medication against OAB syndrome; TM, that is, medication suspected to trigger OAB syndrome.

Adjusting for age and indicators of medical condition, all investigated TM classes showed higher risks for the need of MM with 95% CIs excluding parity, except of class “S” (sensory organs). Class S consists of topical agents for eyes and ears with presumably little or no systemic effect. In other words, women who filled a prescription for one or more TM from ATC class “A” (alimentary tract and metabolism) in the months January to June 2012 had a 40% higher risk of filling a prescription for a MM in the months July to December 2012 (RR of 1.40; 95% CI: 1.29‐1.51; Table 3). ATC class A contains amphotericin B, metronidazole, dexamethasone, and metoclopramide medications, which are suspected to trigger OAB.

Filling prescriptions for class “G” (genitourinary system and sex hormones) and “J” (anti‐infectives for systemic use) caused the highest increase in risk for filling prescriptions for MM (by 109% and 89%, respectively). Filling prescriptions for medication class “C” (cardiovascular system) caused the lowest, but still significant increase in the risk for a MM (by 15%).

In addition to the analysis presented for the full age range of our cohort, we performed the same way of analysis restricted to the 977 886 women ≥65 years old (Table S1). In women ≥65 years old, filling a prescription for TM still held a significantly higher risk for the intake of MM but the adjusted RRs were generally smaller than in the general population. For 6 of 11 medication classes, the RRs were approximately equal to those in the general population. In the older female population, the RRs for filling a prescription for MM were smaller for 5 of 11 medication classes. This effect was possibly owed to the higher prevalence of MM intake in this age group, which was almost twice as high as in the general population (1.20% compared to 0.66%).

When we extended the analysis for the full age range to the more specific medication classes of ATC level 2, the overall result was similar (see results Table 4). Six medication classes on ATC level 2 showed an adjusted RR of >2 with CIs excluding parity. These six classes were “antipruritics including antihistamines, anesthetics, and so on” (D04), “sex hormones and modulators of the genital system” (G03), “antimycotics for systemic use” (J02), “immunostimulants” (L03), “anesthetics” (N01), and “other nervous system drugs” (N07). In other words, filling prescriptions for medication from these classes increased the risk for the need of medication against OAB the most.

**Table 4:** RRs with respect to the need of medication against OAB syndrome (MM) for taking versus not taking any medication from the indicated class (TM ATC level 2)

Medication class ATC level 2		Women without TM^a^		Women with TM^a^			95% CI			95% CI	
		Number	% with MM^b^	Number	% with MM^b^	RR crude	Lower limit	Upper limit	RR adjusted	Lower limit	Upper limit
A01	Stomatological preparations	4 181 033	0.66	4065	1.89	2.87	2.30	3.58	1.97	1.57	2.47
A03	Drugs for functional gastrointestinal disorders	4 152 825	0.65	32 273	1.93	2.96	2.74	3.20	1.63	1.50	1.77
A07	Antidiarrheals, intestinal anti‐inflammatory agents	4 184 830	0.66	268	1.49	2.26	0.85	5.97	1.73	0.65	4.62
B01	Antithrombotic agents	4 185 098	0.66	0	0.00	‐c					
C01	Cardiac therapy	4 185 089	0.66	9	0.00	7.56	0.51	112.64	‐d		
C02	Antihypertensives	4 166 561	0.66	18 537	1.70	2.59	2.32	2.89	1.48	1.32	1.66
C03	Diuretics	4 079 187	0.64	105 911	1.61	2.53	2.41	2.65	1.31	1.24	1.38
C05	Vasoprotectives	4 185 098	0.66	0	0.00	‐c					
C07	Beta‐blocking agents	4 132 519	0.65	52 579	1.53	2.36	2.20	2.53	1.49	1.39	1.61
C09	Agents acting on the renin‐angiotensin system	4 183 063	0.66	2035	1.57	2.38	1.69	3.35	1.56	1.10	2.20
D04	Antipruritics including antihistamines, anesthetics, etc.	4 184 788	0.66	310	1.94	2.93	1.32	6.46	2.35	1.03	5.35
D06	Antibiotics and chemotherapeutics for dermatological use	4 180 811	0.66	4287	1.61	2.44	1.93	3.08	1.94	1.53	2.46
D07	Corticosteroids, dermatological preparations	4 134 299	0.66	50 799	1.08	1.65	1.52	1.80	1.50	1.38	1.64
D10	Antiacne preparations	4 185 098	0.66	0	0.00	‐c					
D11	Other dermatological preparations	4 175 179	0.66	9919	0.92	1.39	1.13	1.70	1.48	1.20	1.82
G01	Gynecological anti‐infectives and antiseptics	4 185 043	0.66	55	0.00	1.35	0.09	21.31	‐d		
G02	Other gynecologicals	4 184 241	0.66	857	0.23	0.35	0.09	1.41	0.37	0.09	1.50
G03	Sex hormones and modulators of the genital system	4 087 949	0.63	97 149	1.89	2.99	2.85	3.14	2.18	2.07	2.28
G04	Urologicals	4 185 097	0.66	1	0.00	‐c					
H02	Corticosteroids for systemic use	4 158 666	0.66	26 432	1.44	2.20	1.99	2.43	1.46	1.32	1.62
J01	Antibacterials for systemic use	4 185 066	0.66	32	3.13	4.72	0.69	32.51	2.10	0.30	14.91
J02	Antimycotics for systemic use	4 170 336	0.66	14 762	1.35	2.06	1.79	2.36	2.20	1.92	2.54
J05	Antivirals for systemic use	4 185 098	0.66	0	0.00	‐c					
L01	Antineoplastic agents	4 184 793	0.66	305	0.98	1.49	0.48	4.58	0.96	0.31	2.98
L02	Endocrine therapy	4 182 457	0.66	2641	1.21	1.83	1.30	2.59	1.44	1.00	2.06
L03	Immunostimulants	4 183 357	0.66	1741	1.95	2.95	2.12	4.12	3.29	2.35	4.61
L04	Immunosuppressants	4 181 508	0.66	3590	1.03	1.56	1.13	2.15	1.14	0.82	1.58
M01	Anti‐inflammatory and antirheumatic products	4 099 829	0.65	85 269	1.34	2.08	1.96	2.20	1.64	1.54	1.74
M02	Topical products for joint and muscular pain	4 185 098	0.66	0	0.00	‐c					
M03	Muscle relaxants	4 180 826	0.66	4272	1.94	2.94	2.38	3.64	1.87	1.50	2.33
M04	Antigout preparations	4 184 934	0.66	164	0.61	0.92	0.13	6.50	0.43	0.06	3.03
N01	Anesthetics	4 166 506	0.65	18 592	2.15	3.28	2.97	3.61	2.20	1.99	2.43
N02	Analgesics	4 180 758	0.66	4340	2.19	3.32	2.72	4.05	1.21	0.99	1.49
N03	Antiepileptics	4 130 103	0.65	54 995	1.87	2.90	2.73	3.08	1.85	1.73	1.97
N04	Anti‐Parkinson drugs	4 149 164	0.65	35 934	2.26	3.49	3.26	3.74	1.82	1.69	1.96
N05	Psycholeptics	4 109 015	0.65	76 083	1.44	2.22	2.09	2.35	1.25	1.17	1.33
N06	Psychoanaleptics	3 819 474	0.58	365 624	1.51	2.60	2.53	2.68	1.71	1.65	1.76
N07	Other nervous system	4 183 738	0.66	1360	1.47	2.22	1.44	3.44	2.05	1.32	3.18
P01	Antiprotozoals	4 163 505	0.66	21 593	1.29	1.96	1.75	2.21	1.89	1.68	2.13
R01	Nasal preparations including decongestants	4 185 098	0.66	0	0.00	‐c					
R06	Antihistamines for systemic use	3 992 177	0.64	192 921	1.02	1.59	1.51	1.66	1.65	1.57	1.73
S01	Ophthalmologicals	4 180 249	0.66	4849	1.48	2.25	1.79	2.83	1.11	0.88	1.40
S02	Otologicals	4 185 098	0.66	0	0.00	‐c					
S03	Ophthalmological and ontological preparations	4 185 098	0.66	0	0.00	‐c					

*Note*: Shown are crude and adjusted relative risks including 95% CI, see Statistical Analysis section for list of adjustment variables;

Abbreviations: CI, confidence interval; MM, marker medication; OAB, overactive bladder; RRs, relative risks; TM, trigger medication.

a Within first half of 2012;

b Within second half of 2012; MM, that is, anticholinergic medication against OAB syndrome; TM, that is, medication suspected to trigger OAB syndrome;

c No RRs calculated due to absence of women with TM.

d No adjusted RR calculated due to separation; crude RR bias corrected.

Filling prescriptions for medication from seven classes on ATC level 2 did not show a RR of statistically significance with respect to the need of medication against OAB syndrome. These seven classes were “antidiarrheals, intestinal anti‐inflammatory/anti‐infective agents” (A07), “other gynecologicals” (G02), antibacterials for systemic use” (J01), antineoplastic agents (L01), “immunosuppressants” (L04), antigout preparations (M04), and “ophthalmologicals” (S01).

## 3 DISCUSSION

This study found firstly that the use of prescribed anticholinergic drugs to treat OAB syndrome increased significantly over a recent 5‐year period in the study population. Secondly, the results confirmed that the initiation of certain TM, most importantly sex hormones and anti‐infectives, carries an increased risk to develop OAB syndrome in women who previously had not used either group of medication. These results validate suspected substances as “TM” for OAB in a large population‐wide data set.

In Austria, the filling of prescriptions for anticholinergic drugs to treat OAB syndrome increased from an average of 0.66% in 2009 to 0.84% in 2012, in other words from 1 in 151 to 1 in 123 women. The increase of fillings of prescriptions for OAB medication indicates an increase of OAB in the investigated population and underpins the relevance of our research question.

This is relevant because the anticholinergic burden is considered a major health issue and carries risks of cognitive impairment and deleterious slips and falls.^28^

One possible explanation for this increase in prescriptions for anticholinergic drugs is the fact that all types of incontinence are more common with age and obesity,^29^ and the burden of these conditions does increase with current demographic trends. Another possible explanation would be an increasing awareness for the medical problem of urinary incontinence in both, women and caregivers. It has been shown before that despite increasing evidence of adverse outcomes, the proportion of older people who are prescribed anticholinergic medications and the proportion with a high anticholinergic exposure have increased over time.^30^

Our large population‐based cohort study shows increased RRs with respect to the need of medication against OAB syndrome for nearly all substance classes in which 188 suspected trigger substances are listed in the ATC classification system. This corroborates the role of these 188 substances as suspected TM for OAB syndrome. In most cases, suspected TMs are not the only substances listed in the respective ATC group. The fact that few suspected substances in one class caused a statistical effect for the entire class strengthens the assumption that our list of TM is valid. This is especially true for medication of classes “antipruritics including antihistamines and so on” (D04), “sex hormones and modulators of the genital system” (G03), “antimycotics for systemic use” (J02), “immunostimulants” (L03), “anesthetics” (N01), and “other nervous system drugs” (N07). These results corroborate the demand to investigate patients' medication use as part of the diagnostic work up for women with OAB syndrome.^29^

We computed the list of 188 substances that we defined as TM based on a thorough review of the existing literature and a search of “Clinical Pharmacology,” an online pharmacologic database. We acknowledge the fact that this list might not be complete and that more substances, especially from the same class, for example, imipenem in addition to meropenem, rivastigmine or galantamine in addition to donepezil might be TMs. We have only analyzed substances that to date have been suspected to cause or worsen OAB. This study did not set out to detect new substances which might act as TM for OAB syndrome. This study set out to confirm in a large, population‐based cohort that suspected substances indeed have to be considered as increasing the risk for OAB syndrome.

Polymedication is recognized as a major health problem and our study adds to the importance of taking analyzing medication intake in patients. In this way, our study has the potential to raise the awareness of pharmacovigilance in caregivers concerned with urinary incontinence.

We statistically tested 44 out of 94 of all classes of medication on ATC level 2. We did not “screen” our database for new TM. To test or “screen” the more than 1400 known medical substances for yet unknown TM would require a database with even more than the 4+ million data sets which were available for this study.

One strength of this study is the fact that with 4 185 098 subjects, it is the largest study on this topic to date. Previous studies were based on smaller cohorts or populations.^4^, ^5^

Another strength of this study is the fact that by controlling for hospital discharge diagnoses we were able to reduce the risk of confounding bias in our analysis. We corrected for 43 ICD codes including medical conditions like mood disorders, diabetes mellitus, and dementia, which are all known to be a risk factor for urinary incontinence.

We had to make general assumptions for the period of intake of MMs regarding our prevalence estimate, as no individual intake and compliance data were available. Our approach to investigate the influence of TM on filling prescriptions for MM is not based on individual time paths of prescription fillings. It is rather based on a partition of the time line for all individuals into a “harvesting period” for the covariables including TM (ie, the first half of 2012) and a “target period” for determining the OAB status of the women (ie, the second half of 2012). More sophisticated analyses could be applied in order to make full use of the information from the distance in time between utilizing TM and MM. But our approach clearly separates TM utilization and MM utilization, and thus is fully valid.

There are limitations to our study. First, we based the diagnosis “OAB syndrome” on the fact that women filled a prescription for anticholinergic medication rather than on a clinical examination. Thus, our conclusions refer to the consequences of filling prescriptions for TM on the filling of prescriptions for anticholinergic medication rather than on the influence of true medication intake on the incidence of OAB. However, the substances trospium, oxybutynin, tolterodine, and solifenacin are almost exclusively prescribed to treat OAB, and it seems fair to assume that filling a prescription for anticholinergic medication is a reliable proxy for the presence of OAB syndrome.

On the other hand, women who suffer from OAB but do not take anticholinergic drugs against it could not be detected in this study. Anticholinergics that were not available in Austria during the study period were darifenacin, desfesoterodine, emepronium, fesoterodine, flavoxate, meladrazine, mirabegron, propiverine, and terodiline. Also, we do not know the proportion of women who fill a prescription for medication but then do not take it or do not take the prescribed medication appropriately. However, a significant proportion of noncompliance would only increase the observed effect of TM necessitating the prescription for anticholinergic medication and would thus not change our conclusion.

Our assessment of OAB was solely based on the utilization of medication to treat OAB. There are other ways to treat OAB than taking medication, and our approach may have missed OAB cases in which women chose to be treated by physical therapy or onabotulinum toxin A injections only.

However, this restriction is unlikely to exaggerate the effects of TM on the occurrence of OAB, on the contrary; we rather assume that the effects are underestimated.

## 4 CONCLUSION

In women, the utilization of certain prescription medication is a significant risk factor for the development of OAB syndrome and the need to take anticholinergic medication, thereby contributing to anticholinergic burden.

## CONFLICT OF INTEREST

The authors declare no conflict of interest.

## Supporting information


**Table S1** **.** Relative risks (RR) with respect to the need of medication against overactive bladder syndrome (marker medication) for taking versus not taking any medication from the indicated class (trigger medication, ATC level 1) for women aged ≥ 65 years of age

**File S1** **.** STROBE checklist of items that should be included in reports of cohort studies
